# Environmental monitoring and equipment sanitation to mitigate *Listeria* risks in a soil-based controlled environment agriculture system

**DOI:** 10.1128/aem.01396-25

**Published:** 2025-11-20

**Authors:** María Ayala-San Nicolás, Pilar Truchado, Ana Allende, Natalia Hernández, Silvia Andújar, Juan Antonio Tudela, María Isabel Gil

**Affiliations:** 1 Research Group on Microbiology and Quality of Fruit and Vegetables, CEBAS–CSIC 54424 , Murcia, Spain; University of Georgia Center for Food Safety, Griffin, Georgia, USA

**Keywords:** *Listeria monocytogenes*, controlled environment agriculture (CEA), environmental monitoring, leafy greens, cross-contamination, whole genome sequencing

## Abstract

Controlled environment agriculture (CEA) offers protection from external contaminants but introduces unique food safety challenges, particularly related to equipment sanitation. This study evaluated the occurrence and persistence of *Listeria monocytogenes* and related species in a commercial soil-based CEA facility through a 1-year environmental monitoring program (EMP). A total of 169 samples, including baby leaves, soil, water, and swabs from equipment surfaces, were collected across three visits. *L. monocytogenes* was detected in only one sample (0.59% prevalence), whereas *Listeria innocua* was isolated from harvesting crates and structural surfaces (3/169). Molecular confirmation and characterization were conducted using PCR (*hly, iap, sigB*), matrix-assisted laser desorption/ionization time-of-flight mass spectrometry (MALDI-TOF MS), and whole genome sequencing (WGS). To assess the potential for cross-contamination, *L. innocua* isolates were used for inoculation studies on reusable plastic crates. Viable cells survived up to 24 h post-inoculation despite water rinsing under 25°C and 60%–70% relative humidity. These findings suggest a low risk of *L. monocytogenes* contamination in this soil-based CEA system but underscore the importance of targeted sanitation, particularly for harvesting equipment, to prevent harborage and transfer of *Listeria* spp.

**IMPORTANCE:**

Controlled environment agriculture (CEA) is a rapidly expanding approach to producing fresh food year-round with greater resource efficiency. However, it presents unique challenges for managing foodborne pathogens. This study demonstrates that in this soil-based indoor system, contamination risks persist, particularly via inadequately cleaned harvesting equipment. The boot cover finding is interpreted as an indicator of environmental contamination, but it does not constitute evidence of footwear-to-crop transfer risk. Although *Listeria monocytogenes* was rarely detected, related species like *Listeria innocua* persisted on reusable plastic crates and structural surfaces, highlighting weaknesses in current sanitation protocols. The use of boot covers as environmental monitoring tools proved valuable for detecting contamination sources. These findings underscore the need for tailored sanitation strategies and comprehensive environmental monitoring programs to enhance food safety in CEA systems and prevent pathogen harborage and spread.

## INTRODUCTION

Controlled environment agriculture (CEA) includes crop production systems that integrate horticultural practices and advanced agricultural methods to optimize yields and enable year-round cultivation. In soil-based CEA, crops are protected from adverse environmental conditions, such as extreme weather, whereas water and nutrient delivery are precisely managed through sprinkler fertilization. Many leafy vegetable species allow multiple successive harvests (“cut and regrowth”). In this facility, the number of cuts per planting varied with the production period and environmental setpoints (e.g., temperature and light), typically ranging from two to six, depending on the species. Due to seasonal temperature variations, harvest frequency is higher in winter with up to 5–6 harvests, whereas in summer, elevated temperatures reduce this to only about 2 harvests (1). These continuous, high-production cycles result in frequent contact between harvesting equipment and produce, increasing the risk of foodborne pathogens transfer, including *L. monocytogenes*. Controlling pathogen entry and minimizing transmission has been identified as a key research priority for CEA operations (2).

The risk of *L. monocytogenes* contamination in soil-based CEA facilities, particularly those producing leafy greens, is a major concern due to the pathogen’s persistence in soil and on surfaces. Once established in the production environment, elimination becomes challenging. Its ability to adhere to surfaces enhances its resistance to cleaning and sanitation, as demonstrated in cantaloupe contamination studies (3). To mitigate risk and ensure the safety of leafy greens, effective environmental monitoring (EM) sampling is essential. Effective monitoring requires a systematic approach, prioritizing *L. monocytogenes* detection while using *Listeria* spp. as an indicator of overall contamination (4, 5).

Research on the transfer of human pathogens during postharvest operations has identified multiple crop-contact points that can contribute to contamination, such as surfaces (6). If these surfaces harbor human pathogens, they can facilitate transfer to crops during harvesting. For example, *L. monocytogenes* has been detected on harvesting equipment used for iceberg lettuce, with WGS confirming a match to contaminated salads (7). Similarly, swab samples from cutting blades and reusable crates have tested positive for *L. monocytogenes* (8). Although reusable plastic crates have not been directly linked to foodborne outbreaks, indirect evidence suggests a potential risk when hygiene practices are inadequate (9). Contamination may also occur due to harvesting machine movements displacing soil onto crates, where soil and crop residue, potential reservoirs for *Listeria*, can accumulate on product contact surfaces (6).

Additionally, surface roughness plays a critical role in cleaning and biofilm removal. Rough or damaged surfaces create ideal conditions for bacterial adhesion and biofilm formation (10). These irregularities can shield bacteria from sanitizers. Physical forces such as scrubbing or high-pressure washing are essential to disrupt biofilms and improve cleaning effectiveness. Maintaining smooth, undamaged surfaces is critical to minimizing bacterial attachment (6). Harvesting equipment, including reusable plastic crates, often lacks regular deep cleaning, increasing contamination risks from soil, plant debris, and bacteria (11). Implementing scheduled deep cleaning at appropriate intervals is indispensable to ensure sanitation of hard-to-reach areas and to support safe reuse practices, preventing microbiological cross-contamination.

In this study, a soil-based CEA facility employing a regrowth system, where multiple harvests are obtained from the same seed, was sampled three times over a 1-year cultivation period. The objectives were to (i) identify potential contamination sources of *L. monocytogenes*, (ii) characterize *Listeria* isolates to assess persistence and diversity, and (iii) evaluate the effectiveness of current cleaning practices for reusable harvest crates.

## RESULTS

### Presence of *L. monocytogenes/Listeria* spp. in the soil-based CEA facility

A total of 169 samples were collected from 15 sampling sites. From Fraser-positive samples, 219 isolates were recovered, with the majority from MOX selective media. Of these, only 42 tested positive for at least one of the targeted genes. Twenty-three representative isolates from various sites, including harvesting crates, structural columns, boot covers, and the leaf vacuum machine, were selected for MALDI-TOF MS analysis. Based on the MALDI-TOF identification, 13 isolates were identified with high confidence as *Listeria* species, matching the best reference species (Table S1). In EM1, *L. innocua* was identified in harvesting crates (before and after vacuum cooling) and on structural columns. In EM2, *Listeria fleichmannii*, *Listeria grayi,* and *Listeria aquatica* were identified from crates (both pre- and post-vacuum cooling), with low confidence scores. Additionally, *Enterococcus faecalis* was identified from the leaf vacuum machine. In EM3, *L. monocytogenes* was the only *Listeria* species identified in the boot covers.

### Genomic characterization of *L. monocytogenes/Listeria* spp. isolates

WGS was performed on eight selected isolates using Illumina technology, known for its high accuracy, sensitivity, and throughput. Sequencing quality was assessed using standard metrics, including total bases, read count, GC content, and Q20/Q30 scores, which indicate base-calling confidence (Table S2). The data showed high sequencing quality, with sufficient depth for genomic analysis. For variant detection, samples with Q30 values above 90% were considered highly reliable. Filtered reads were mapped to the *L. monocytogenes* reference genome (GCF_000196035.1), and assembled sequences were compared against the reference database. Some isolates were identified as different *Listeria* species or remained unclassified, typically due to low mapping percentages. According to WGS results, *L. innocua* was the only confirmed species in harvesting crates (before and after cooling) and on structural columns. *Listeria* species previously identified by MALDI-TOF MS with low-confidence scores (30%) could not be confirmed by WGS (Table 1). To further investigate the identity of the isolates that did not align significantly with the *L. monocytogenes* reference genome, raw reads were first assembled *de novo* using SPAdes, and the resulting contigs were subsequently analyzed with Kraken2 for taxonomic assignment. One isolate was confidently identified as *Bacillus circulans*, whereas the remaining isolates were assigned to the Enterobacteriaceae family, without resolution to genus or species level. These results indicate that the non-matching isolates likely correspond to non-*Listeria* bacteria.

**TABLE 1:** Identification of isolates in the soil–based CEA facility across three environmental monitoring events (EM1, EM2, and EM3) from various sampling points with the detection of genes *sigB*, *iap*, and *hly*, presumptive identification by MALDI–TOF MS, and confirmation sequencing by whole genome sequencing (WGS) analysis^*a*^

EM	Sampling point	Detected genes			MALDI–TOF	WGS
		*sigB*	*iap*	*hly*		
1	Harvesting crates	Positive	Positive	Negative	*L. innocua*	*L. innocua*
	Harvesting crates after vacuum	Positive	Positive	Negative	*L. innocua*	*L. innocua*
	Columns	Positive	Positive	Negative	*L. innocua*	*L. innocua*
	Boot covers	Negative	Positive	Negative	*L. aquatica*	*Bacillus circulans*
2	Harvesting crates	Negative	Positive	Negative	*L. fleichmannii*	NSM
	Harvesting crates after vacuum	Negative	Positive	Negative	*L. grayi*	NSM
	Harvesting crates after vacuum	Negative	Positive	Negative	*L. aquatica*	NSM
3	Boot covers	Positive	Positive	Positive	*L. monocytogenes*	*L. monocytogenes*

^*a*^ NSM, no significant matching.

The presumptive *L. monocytogenes* strain from the boot covers (EM3) was further genotyped using multilocus sequence typing (MLST), based on seven housekeeping genes (*abcZ, bglA, cat, dapE, dat, ldh,* and *lhkA*) (12), via the BIGSdb-Pasteur v1.36.7 web server (https://bigsdb.pasteur.fr/) (13). The isolate was assigned to Lineage II, Sequence Type (ST) 155, and Clonal Complex (CC) 155. Core genome MLST (cgMLST) further classified it as core genome type (CT) 29,464. These results confirmed the presence of *L. monocytogenes* in only one sample (0.59%, 1/169), detected on a boot cover during EM3 (Table 1). No *L. monocytogenes* was identified in other sample types, including surfaces, soil, crops, or water.

### Cleaning and sanitizing practices for harvesting crates

**Fig 1:**
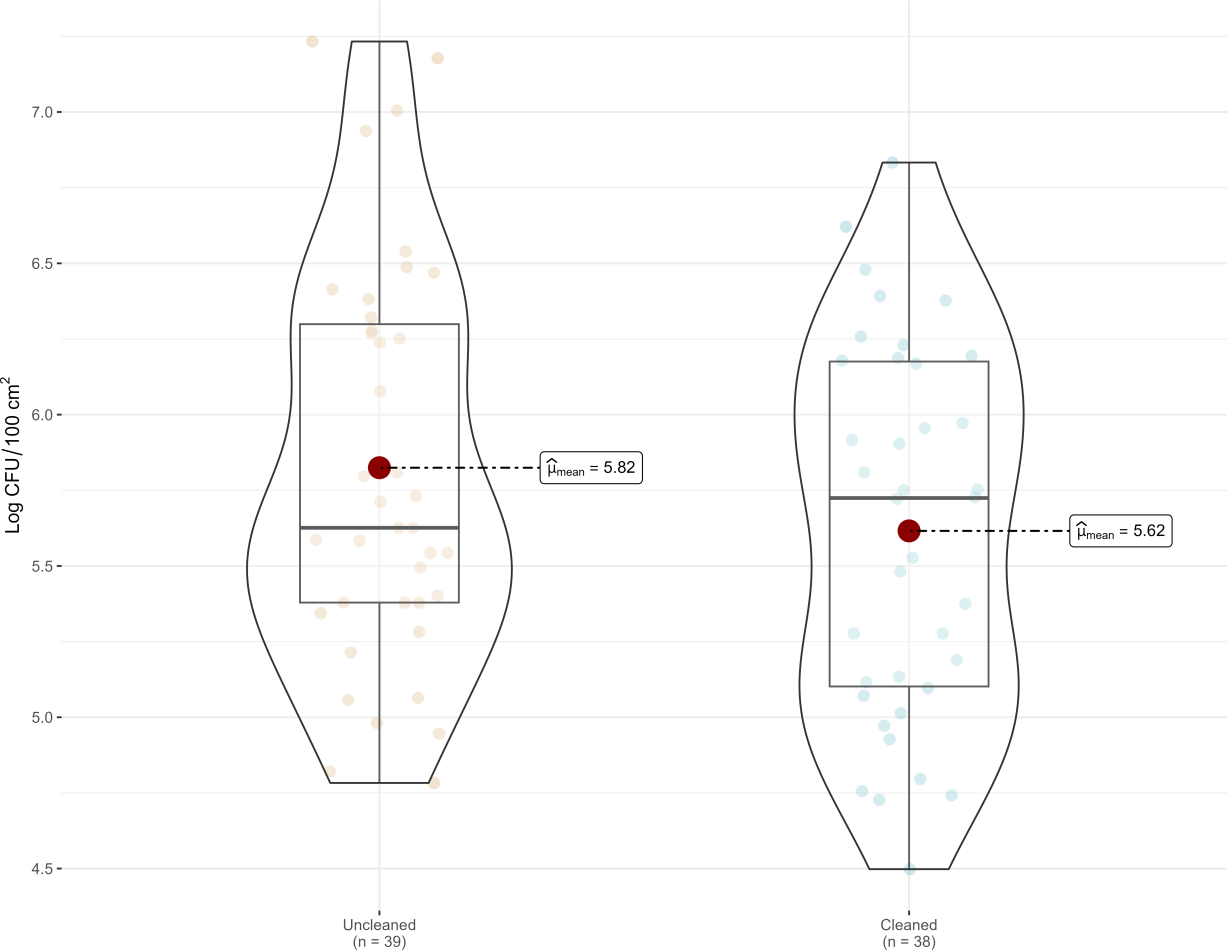
Box plot representing the microbial load, measured as aerobic plate count (APC), in uncleaned and cleaned harvesting crates, based on the mean of two trials (Trial 1: May 15 and Trial 2: July 2). The mean is indicated by a dot, representing the mean average microbial load of 39 uncleaned crates and 38 cleaned crates. The horizontal line inside each box represents the median microbial load (50th percentile).

Analysis revealed that reusable plastic crates exhibited elevated microbial loads, with high aerobic plate counts (APC) detected both before and after cleaning (Fig. 1). The highest APC levels were observed on surfaces with visible soil before cleaning. Mean APC levels were 5.82 log CFU/crate (SD = 0.66) in unclean crates and 5.62 log CFU/crate (SD = 0.62) in cleaned crates. A Welch’s *t*-test indicated that the difference was not statistically significant, *t*(74.88) = 1.44, *P* < 0.15, d = 0.32. These results suggest that although the cleaning protocol effectively removed visible soil, it did not significantly reduce microbial loads. Swab samples tested negative for *L. monocytogenes* and *Listeria* spp., both before and after cleaning during trials conducted in May and July. An additional trial was conducted in November 2024 under lower temperature and higher relative humidity conditions. In this trial, 60 unclean reusable harvesting crates were sampled, and none tested positive for *Listeria* spp.

### Contamination risk of harvesting crates and surface cleanability

**Fig 2:**
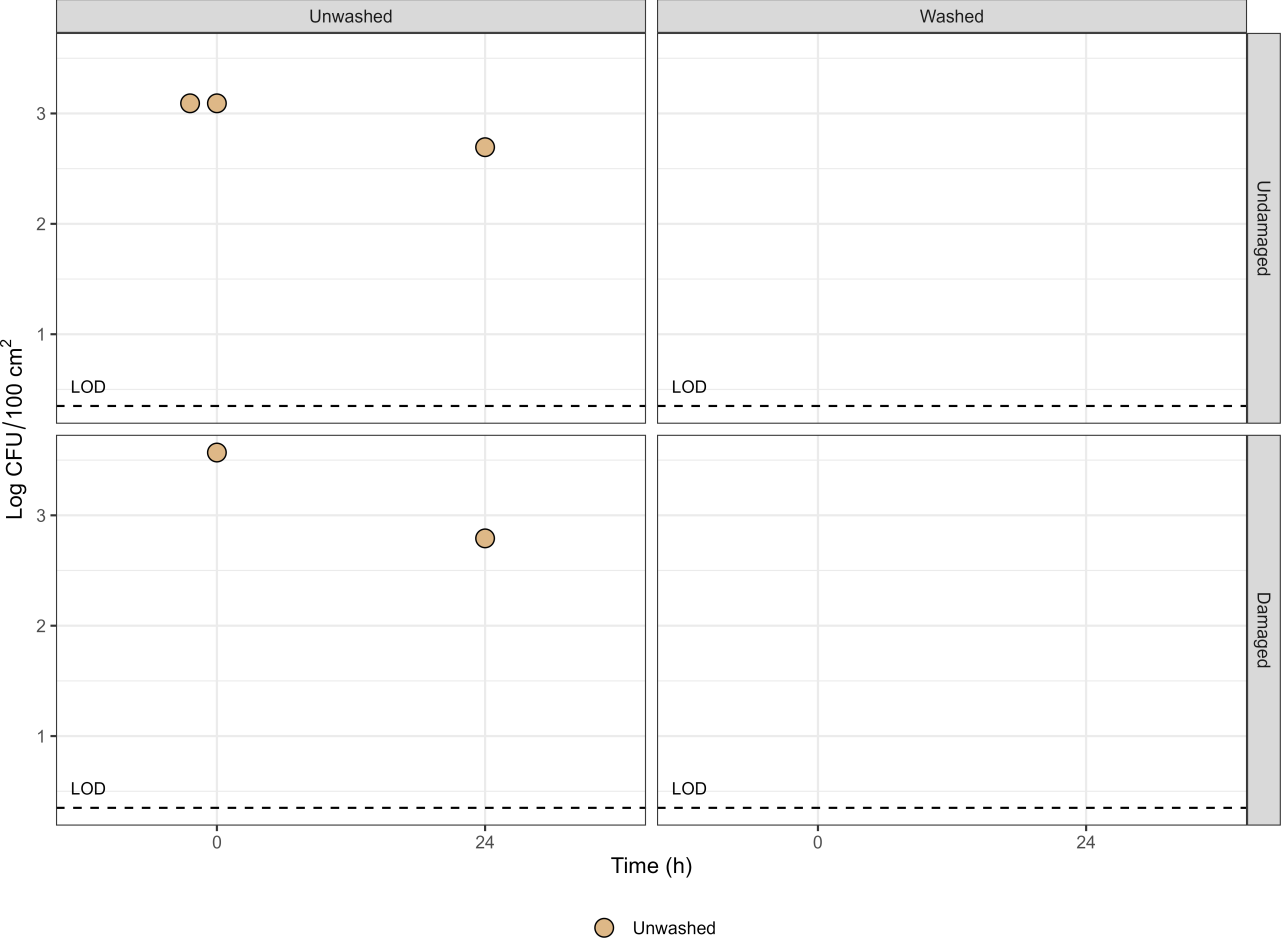
Enumeration of *L. innocua* on unwashed and water-washed polypropylene coupons with undamaged or damaged surfaces at T0 and T24 (24 h storage at 25°C, 60%–70% RH). Values are shown as log CFU/100 cm². Detected replicates (*N* = 4) (> LOD = 0.35) per condition are plotted in the graphs.

No significant differences in *L. innocua* recovery were observed between swabbing and sonication, regardless of surface type. However, *L. innocua* survival on clean coupons, whether undamaged or damaged, showed considerable variability. On undamaged surfaces, *L. innocua* was detected in two out of the four analyzed samples, with counts reaching 3.09 log CFU/100 cm² (mean of 1.55 log CFU/100 cm²). On rough, damaged surfaces, *L. innocua* was recovered from only one of the four samples, with a count of 3.57 log CFU/100 cm² and a mean of 0.89 log CFU/100 cm² (Fig. 2). After 24 h (before washing), mean counts were similar for undamaged and damaged surfaces (0.67 and 0.70 log CFU/100 cm², respectively). Regardless of surface roughness, no viable bacteria were detected after washing, either immediately after inoculation (T0) or following 24 h of storage at 25°C and 60% RH (T24).

**Fig 3:**
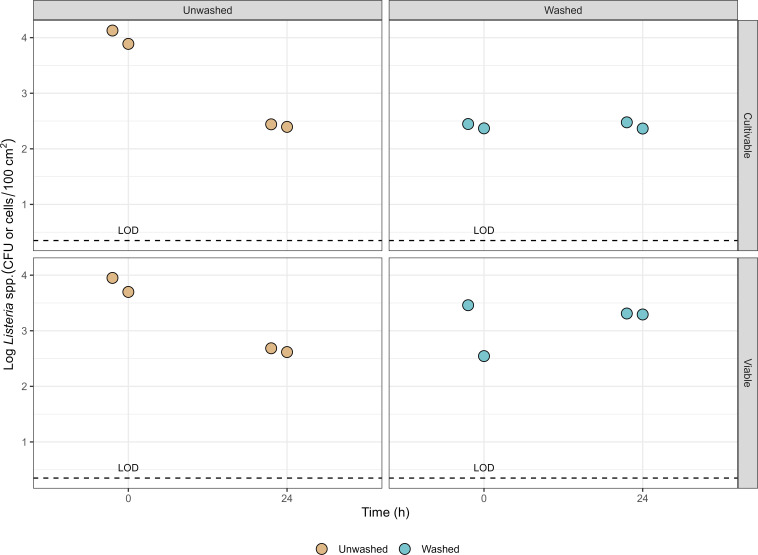
Survival of *L. innocua* as culturable bacteria (log CFU/100 cm²) and viable cells (cells/100 cm²) on unwashed and water-washed harvest crates at T0 and T24 (24 h storage at 25°C, 60%–70% RH). Detected replicates (*n* = 2) (> LOD = 0.35) per condition are plotted in the graphs.

The persistence of *L. innocua* was also evaluated on uncleaned harvesting crates, where organic matter may offer protection. Viability was assessed using both the culture-based method and v-qPCR at T0 and T24, before and after rinsing with water. This aimed to determine whether viable cells survived environmental conditions and rinsing, simulating real-world producer practices. At T0, *L. innocua* counts were 4.0 log CFU/100 cm^2^ (cultivable) and 3.8 log cells/100 cm^2^ (viable). After rinsing, counts decreased to 2.4 log CFU/100 cm^2^ and 3.0 log cells/100 cm^2^. At T24*,* cultivable and viable counts were 2.4 and 2.9 log CFU or cells per 100 cm^2^, respectively, while after rinsing, bacteria persisted, with 2.4 log CFU and 3.3 log cells/100 cm^2^, respectively, detected (Fig. 3).

## DISCUSSION

### Environmental distribution of *L. monocytogenes/Listeria* spp. and genetic lineage

The environmental monitoring (EM) conducted in the soil-based CEA system aimed to identify potential pathogen reservoirs across soil, surfaces, crops, and water. This study underscores the complexity of sampling large-scale greenhouse environments, particularly due to the diversity of sample types and locations. A central challenge in EM is determining appropriate sample sizes that balance representativeness with practical feasibility, especially in terms of replication. To enhance representativeness, the number of surface replicates was increased from *n*  =  3 in EM1 and EM2 to *n*  =  5 in EM3. This expanded sampling effort contributed to the detection of *L. monocytogenes*, which was found in only one sample, boot covers, indicating low prevalence in the facility overall. The isolate was recovered during EM3, when the sampling intensity was greatest, reinforcing the value of increased replication in detecting sporadic contamination.

Boot covers proved to be particularly effective as sampling tools, consistent with previous findings (14). Their ability to collect material from a broad area, including soil, water residues, and plant debris, makes them a practical and efficient method for aggregate environmental monitoring in indoor cultivation systems. Our findings support their utility in soil-based CEA operations, where direct sampling of multiple surfaces can be logistically challenging.

Previous studies have shown that the presence of *L. innocua* in foods contaminated with *L. monocytogenes* can reduce the likelihood of detecting *L. monocytogenes* (15). These studies suggest that coexisting *L. innocua* strains may suppress the growth of *L. monocytogenes* during selective enrichment. Additionally, previous studies have shown that MOX has lower specificity and sensitivity than other chromogenic agars for detecting *Listeria* (16, 17). This limitation was also observed in our study, as MOX supported the growth of other genera, including *Enterococcus* spp. (e.g., *E. faecalis*), which were not detected on Oxoid chromogenic listeria agar (OCLA). WGS analysis in our study confirmed that the isolate testing positive for the *hly* gene by PCR was indeed *L. monocytogenes*, whereas those positive only for *iap* and *sigB* corresponded to other *Listeria* species. These results underscore the importance of combining selective media with molecular and genomic tools to enhance detection specificity and ensure accurate species-level identification.

The *L. monocytogenes* isolate recovered from the boot cover sample was classified as Sequence Type (ST) 155, a member of Lineage II—predominantly associated with food and environmental sources rather than clinical cases—confirming its environmental origin and alignment with strains commonly found in non-clinical settings (18). ST155 has been identified in a variety of food matrices, processing environments, and natural reservoirs (19, 20). It has also been detected in mixed vegetables (21) and in agricultural and natural environments such as soil, water, plant material, livestock, and farm settings (22). ST155 is known for its capacity to persist on food processing surfaces, drains, and equipment, often through biofilm formation (23, 24). Although lineage II ST155 is considered hypovirulent and is less commonly linked to severe human listeriosis outbreaks, its presence in food production environments still represents a significant food safety concern (25). Our detection of this genotype in a boot cover sample reinforces its environmental persistence and potential role in contamination pathways in this CEA system. Its persistence highlights the need for robust hygiene protocols, ongoing environmental monitoring, and targeted preventive measures to minimize contamination and prevent transmission along the food chain (26, 27).

### Risk factors of cleaning efficacy in harvesting equipment

From our visits to the CEA facility, we observed an increased risk of contamination associated with mechanized cultivation and harvesting operations. Harvested tender leaf products may come into direct contact with soil in many growing and harvesting processes, including vacuum cooling. Harvest crates were sampled after vacuum cooling; this step was considered quality management, not a cleaning intervention. Enhanced cleaning and sanitation procedures should be implemented after events and conditions that contribute to greater soil accumulation on equipment (6). Although no soil samples tested positive for *L. monocytogenes*, its detection on boot covers underscores the potential role of soil as a contamination reservoir. The presence of *L. monocytogenes* on boot covers suggests that soil can act as a vector for pathogen transfers to produce, particularly through intensive soil movement, such as the passage of leaf vacuum machines over crop or splashes from sprinkler irrigation. In addition, detection of *L. monocytogenes* on a boot cover, at the same time as negative results from spot surface swabs, indicates that sampling with broader coverage along walking paths can be more effective than point swabbing for detecting sporadic contamination. This supports the use of footwear sampling as an early-warning method in soil-based CEA, particularly along high-traffic pathways and where the soil meets the facility. During harvesting, product contact surfaces, such as reusable plastic crates used for produce collection, frequently accumulate soil and crop residue. The results showed that surfaces remain potential harborage sites and may contaminate harvested products, especially baby leaves, which have large surface contact areas. Therefore, proper maintenance and regular inspection of reusable plastic crates are essential for compliance with food safety standards. Organic materials can accumulate on food contact surfaces throughout the operational day, making hygiene practices both critical and challenging (28). A thorough water rinse of food contact surfaces before applying a sanitizer is essential for effective disinfection.

Surface condition also plays a critical role in microbial adhesion and persistence. Rough or damaged areas in plastic crates may serve as pathogen reservoirs, facilitating the transfer of microorganisms to produce during vacuum cooling and transport (29). In this study, *L. innocua* was effectively removed from clean crate surface coupons, both smooth and rough, by rinsing with water. However, cracks and material deterioration increase the risk of bacterial retention, particularly when organic matter is present. Previous research describes a two-phase bacterial adhesion process: initial reversible attachment followed by stronger binding within minutes (3). Although loosely attached cells were removed by rinsing, biofilm formation, an important survival mechanism not assessed here, can allow bacteria to persist even under cleaning regimes. Biofilms shield cells from environmental stressors and cleaning agents through extracellular polymeric matrices, increasing resistance (30).

Contaminated surfaces with minimal food residues can support larger populations of *L. monocytogenes* (31), especially when defects such as scratches or cracks are present (32). In our study, *L. innocua* persisted on uncleaned crates for at least 24 h post-inoculation and remained detectable after rinsing, confirming that the presence of organic matter strongly influenced bacterial survival and may limit the effectiveness of simple rinsing. Although our experimental rinse step used only water, routine crate cleaning at the facility includes an alkaline detergent in a cleaning tunnel. A full comparison with commercial sanitation (detergent ± sanitizer) was beyond the scope of this work and is a priority for future studies, particularly to quantify sanitizer performance on soiled surfaces.

Moreover, aerobic plate count (APC) levels remained largely unchanged, likely due to the lack of process optimization. These findings are consistent with previous research showing that cleaning alone is marginally effective in reducing microbial loads, and the inclusion of sanitizers is necessary for optimal microbial reduction (33).

To improve sanitation monitoring, future protocols should incorporate both rapid and traditional microbiological methods to assess the effectiveness of cleaning and sanitation (34). Indicator organisms that are both culturable and quantifiable, such as *Listeria* spp., *Enterobacteriaceae*, and APCs, provide more reliable data for tracking cleaning and sanitation of harvesting equipment across harvesting events or commodity lots. Importantly, the persistence of *L. innocua* detected through culture-independent methods emphasizes the value of incorporating molecular detection tools alongside traditional culturing to detect viable but non-culturable (VBNC) state in food contact surfaces. In the present study, bacterial viability was assessed to determine whether *L. innocua* entered a viable but non-culturable (VBNC) state due to exposure to unfavorable environmental conditions during storage, potentially allowing the bacteria to evade standard detection methods (35). Our findings indicate that uncleaned harvesting crates pose a safety risk, as demonstrated by both culture-dependent and culture-independent methods. Moreover, *L. innocua* persisted under the storage conditions to which it was exposed, highlighting the potential for bacterial survival.

Future studies in CEA should employ risk-based, longitudinal EMPs that increase sampling intensity (≥5 replicates per site), standardize and document sampling effort (swab area, footwear route length, and elution volumes), and combine fixed-area swabs with footwear transects as early-warning sampling tools. Large-volume concentration of irrigation/nutrient water, coupled with culture plus qPCR and whole-genome sequencing, will improve sensitivity and enable source tracking of *L. monocytogenes*. Prevalence and detection should be reported as positives/total with exact 95% CIs and analyzed using models that include sampling effort as a covariate; sensitivity analyses (e.g., down-sampling) should quantify the impact of replicate number. Sanitation effectiveness for reusable containers and harvest equipment should be validated with quantitative log-reduction targets and post-sanitation re-sampling. These measures directly address limitations of our study (sampling intensity, sampling-unit comparability, limited water volumes, and temporal coverage) and provide a framework to resolve contamination sources, persistence, and distribution in CEA facilities.

### Conclusions

This study demonstrates that an EM sampling plan can effectively identify contamination sources and support corrective actions in CEA facilities. Over a year of monitoring leafy green greenhouses, *L. monocytogenes* was found at a very low prevalence, despite the intensive production environment and the potential for contamination transfer from harvesting equipment. *L. monocytogenes* was detected in only one boot cover sample, underscoring the utility of boot covers as a representative, practical, and effective tool for large surface areas. The evaluation of harvesting crates and cleaning practices revealed challenges in improving commercial sanitation protocols. Cleaning and sanitation of harvesting equipment should be optimized to enhance effectiveness. Cleaning was only marginally effective in reducing microbial load in harvesting crate tunnels. Additionally, *L. innocua* persisted longer on uncleaned surfaces than on cleaned ones, increasing the risk of cross-contamination if leafy greens come into contact with contaminated crates. Implementing a robust environmental monitoring program is essential for reducing contamination risk in CEA facilities and ensuring compliance with food safety regulations. Such programs help mitigate potential hazards in the production environment and contribute to the overall safety of fresh produce. We can summarize our findings as recommendations for future studies to adopt risk-based, longitudinal EMP designs that (i) increase sampling intensity and spatial coverage, (ii) standardize and document sampling effort, and (iii) pair culture with molecular and genomic tools to resolve sources and routes of *L. monocytogenes* in CEA.

## MATERIALS AND METHODS

### Sample collection

This study was conducted in a soil-based CEA system consisting of a 36-hectare farm with 30 greenhouses of 1 hectare each plus ~6 ha of support/infrastructure (water reservoirs, roads/tracks, service yards, packhouse/buildings). The farm was dedicated to cultivating baby leaves under commercial agricultural practices involving multiple regrowth cycles. Environmental monitoring (EM) sampling was performed three times over 1 year, conducting samplings in May 2023 (EM1), October 2023 (EM2), and April 2024 (EM3). A total of 169 samples were collected from 15 selected sampling points, including produce, irrigation water, soil, amendments, equipment, and harvesting crates (Table 2). For surface swabbing, three replicate swabs were collected per surface during EM1 and EM2, whereas in EM3, five replicate swabs were collected per surface. Sterile sponge swabs (3M Sponge Stick Pack Of 5X20 SSL100), moistened with sterile water, were used to sample surfaces such as harvester blades, conveyor belts, structural columns, and the suction inlet of the leaf vacuum machine. The vacuum machine, which passes over crops after each harvest cycle to remove residual leaves, was swabbed along with the wheels of the harvester, vacuum machines, and the reusable plastic crates. Each surface was thoroughly swabbed, ensuring complete coverage. Sponges were then immersed in sterile bags filled with 100 mL of Half Fraser broth (Scharlab, Barcelona, Spain). A locally common sheep manure-based compost that includes stabilized plant fibers/pruning residues was used as a soil amendment. For soil and soil amendments, 25 g samples were collected in sterile bags containing 225 mL of Half Fraser broth, with three replicates in EM1 and EM2, increasing to five in EM3. Additionally, boot covers (*n* = 3 in EM1 and EM2, and *n* = 5 in EM3) worn during sampling were analyzed as a representative indicator of the greenhouse soil surface. Water samples were collected in each EM from irrigation water at the greenhouse entry point and from the water reservoir. Three replicates of 10 L each were filtered using Modified Moore Swabs (36). Crop samples consisted of five replicates of loose-leaf lettuce in EM1 and EM2 and mizuna in EM3. For each sampling, 25 g of the product was collected directly from the harvester, and an additional 25 g was taken after vacuum cooling. This cooling process was performed at a facility near the greenhouses for all harvested products before shipping to clients. Immediately after the product was collected, 225 mL of Half Fraser broth was added to each sterile sample bag. Samples were then refrigerated in a cooling box with ice packs and transported to the CEBAS-CSIC laboratory, where they were processed within 1 h.

**TABLE 2:** Types of samples, sampling sites, and number of replicates in the three environmental monitoring events (EM1, EM2, and EM3) of the soil–based CEA facility

Types of samples	Sampling sites	Number of replicates
Crop	Harvested product	EM1, EM2, and EM3 (*n* = 5)
	Vacuum-cooled harvested product	
Soil	Soil	EM1 and EM2 (*n* = 3); EM3 (*n* = 5)
	Soil amendments	
Surface	Blade of the harvesting machine	EM1 and EM2 (*n* = 3); EM3 (*n* = 5)
	Boot covers worn during sampling	
	Columns	
	Conveyor belt of the harvesting machine	
	Harvesting crates Harvesting crates after vacuum	
	Leaf vacuum machine	
	Wheel of the harvesting machine	
	Wheel of the vacuum machine	
Water	Greenhouse irrigation water	EM1, EM2, and EM3 (*n* = 3)
	Water reservoir	

### Detection of *L. monocytogenes/Listeria* spp. and characterization of isolates

Samples were processed for the detection of *Listeria* species using a modified version of the ISO 11290-1:2017 method. Briefly, each sample bag containing Half Fraser broth as a primary enrichment was massaged and then incubated at 30°C for 24 ± 1 h. For secondary selective enrichment, 1 mL of the primary enrichment was transferred into 9 mL of Fraser broth (Scharlab, Barcelona, Spain) and incubated at 37°C for 24 ± 1 h. Positive enriched samples were then streaked onto two selective media: OCLA (Oxoid, Thermo Fisher Scientific, Madrid, Spain) and modified Oxford agar (MOX, Oxoid, Thermo Fisher Scientific). OCLA plates were incubated at 37°C for 36–48 h and MOX plates at 30°C for 24–36 h. For each plate, up to five colonies were selected. Thus, sampling points with 3 replicates yielded up to 15 isolates, and those with 5 replicates up to 25 isolates. For each single colony, approximately half was inoculated into Brain Heart Infusion (BHI) broth and incubated at 37°C for 24 ± 1 h. Cultures were then preserved in 30% glycerol at −80°C. The remaining colony was transferred into 100 µL of RNase-free water and boiled at 100°C for 10 min for DNA extraction and then stored at −20°C. PCR analysis was performed, and the resulting products were analyzed via agarose gel electrophoresis at 70 V for 70 min. DNA bands were stained using Red dye staining (Biotium Inc., CA, USA) and visualized with ImageQuant LAS 500 imaging system (GE Healthcare BioSciences AB, Björkgatan, Sweden). The presence of *sigB* and *iap* genes, characteristic of the *Listeria* genus (5, 37), was assessed, along with *hly* as a virulence marker for *L. monocytogenes* (38). Additionally, presumptive isolates were identified using a MALDI-TOF MS system (Bruker Daltonics, Bremen, Germany) with the Bruker Biotyper software platform (39).

### Genomic characterization of isolates

To investigate the relationship among isolates and assess the distribution patterns across sampling sites and contamination routes, WGS and bioinformatics analyses were performed. Sequencing was conducted by Macrogen (South Korea), a globally recognized genomic service provider, ensuring high-precision results. The raw sequencing data were processed through bioinformatics pipelines to achieve comprehensive genomic characterization of the isolates. For bacterial isolation, presumptive *Listeria* isolates were streaked onto BHI agar plates and incubated at 37°C for 24 ± 1 h. Genomic DNA was extracted using the Gentra Puregene Yeast/Bacterial Kit (Qiagen) following the manufacturer’s protocol. DNA concentration and quality were assessed using the Agilent 4150 TapeStation System (Agilent Technologies, Inc.). A total of eight *Listeria* DNA extracts were sent to Macrogen for sequencing. Library preparation was performed using the Nextera DNA XT kit, and sequencing was carried out on the NovaSeq X platform, generating 150 bp paired-end reads. Raw sequencing reads (FASTQ format) were assessed for quality control using FastQC v0.11.7 (40). Adapter sequences and low-quality bases were removed using Trimmomatic v0.38 (41). The filtered reads were then aligned to the reference genome of *L. monocytogenes* (GCF_000196035.1; 2,944,528 bp) using BWA v0.7.17 with the BWA MEM algorithm (42). Duplicate reads were removed using markDuplicates (Picard) (https://broadinstitute.github.io/picard/) to reduce potential bias introduced by PCR amplification. Single-nucleotide polymorphisms (SNPs) and short insertions/deletions (indels) were identified using GATK v4.2.0.0 with HaplotypeCaller (43) and annotation with SnpEff v4.3t (44). Structural variants were detected using Manta v1.6.0 (45), while copy number variations (CNVs) were analyzed with Control FREEC v11.6 (46). Mapping efficiency was assessed by determining the percentage of reads successfully aligned to the reference genomes. High mapping accuracy is crucial for ensuring the reliability of variant detection and subsequent genomic analyses. For bacteria isolates with low mapping efficiency (<10%), the raw sequencing reads (FASTQ files) were reanalyzed using the Galaxy platform v25.0.4.dev0 (47) (https://usegalaxy.org/). Prior to assembly, basic quality control of raw reads was performed using FastQC v0.74 (40), and adapter removal and trimming of low-quality bases were conducted using Trimmomatic v0.39 (41), ensuring high-quality reads for assembly. *De novo* genome assembly was performed using SPAdes v4.2.0 with the operation mode set to assembly and error correction (48). The resulting contigs were subjected to taxonomic classification using Kraken2 v2.1.3 (49) against the Prebuilt RefSeq indexes: core_nt database (version 2024-09-04), which comprises an extensive collection of nucleotide sequences from GenBank, RefSeq, TPA, and PDB.

### Reusable plastic crates and surface cleanability

The cleaning process for reusable plastic crates was conducted at the grower facility located 20 km from the greenhouses. This facility serves as a centralized washing station where uncleaned crates from the greenhouses are transported for automated washing and sanitization before reuse in harvesting and handling operations. The primary objective of the cleaning process was to remove soil and residual cull products. The crates were cleaned in a closed tunnel system following a standardized protocol: an initial washing with potable water to remove loose debris, application of an alkaline detergent (Mip SMA, Ecolab) under medium pressure, as recommended by the manufacturer, to dissolve and remove organic residues, and a final rinse with potable water to eliminate detergent residues. After cleaning, the crates were palletized and redistributed to the CEA facilities for reuse.

The sampling procedure was conducted in May and July, with 20 reusable crates randomly selected from stacked crates before and after cleaning and sanitization. In November, an additional 60 uncleaned reusable crates were sampled. Each crate was individually swabbed, and the swabs were processed as described previously for surface samples. All samples were transported to the laboratory and analyzed within 1 h. Detection of *L. monocytogenes*/*Listeria* spp. contamination was performed following the previously described methodology.

For total microbial load, enumeration was quantified using the aerobic plate count (APC) method. Swabs from Half Fraser medium were serially diluted in tenfold increments, and 100 µL aliquots were plated on plate count agar (PCA) and incubated at 37°C for 24–48 h. The results were expressed as log CFU per cm^2^ of surface area.

### Enumeration and viability of *L. innocua* from surface coupons and crates

The survival of *L. innocua* on polypropylene coupons (9 × 9 cm) was evaluated, with half of the coupons intentionally damaged using an automatic grinder to simulate rough surfaces similar to those found on reusable plastic crates. Coupons were artificially inoculated by spraying them with an *L. innocua* suspension (4 log CFU/mL). Samples were collected at two time points: T0 (1 h post-inoculation) and T24 (24 h of storage at 25°C and 60% relative humidity, RH). At each time point, half of the coupons were rinsed under tap water for 10 seconds immediately before surface sampling to assess the effect of rinsing on *L. innocua* recovery. A water rinse on the cleaned coupons was intended solely to remove loosely attached cells and did not mimic a full sanitation process. The objective of the coupon study was to isolate the effect of surface condition (intact vs. damaged) on cleanability and pathogen attachment without interference from chemical agents. For bacterial recovery methods, two methods were used to assess *L. innocua* persistence on polypropylene surfaces, with two replicates performed for each method: swabbing in which *L. innocua* was recovered using sterile sponges moistened with sterile water to collect the inoculum from the coupons, and sonication/stomaching in which the entire coupon was placed in sterile bags containing 100 mL of Buffered Peptone Water (BPW, 2 g/L), followed by shaking to detach bacteria from the surface.

In addition to the propylene coupons, the survival of *L. innocua* on uncleaned harvesting crates was assessed. The crates were transported to the laboratory and inoculated following the same protocol described for coupons. The *L. innocua* isolates from harvesting crates (stored in BHI containing 30% glycerol at −80°C) were used for inoculum preparation. To prepare the inoculum, 100 µL of the stored culture was transferred to 10 mL of fresh BHI and incubated at 37°C for 72 h. Subsequently, 100 µL of this 72 h culture was added to 5 mL of fresh BHI and incubated at 37°C for 24 h. The resulting culture was centrifuged twice at 3,500 × *g* for 10 min each, and the pellet was resuspended in phosphate-buffered saline (PBS) after each centrifugation. This process yielded an inoculum with a concentration of approximately 8 log CFU/mL, which was then diluted to a target concentration of 4 log CFU/mL, confirmed by plating on OCLA selective media. Under controlled conditions, uncleaned crates were inoculated with 30 mL of *L. innocua* suspension. After 1 h, crates were either swabbed immediately (T0) or stored for 24 h at 25°C and 60% RH (T24) to simulate storage conditions. To assess culturability, *L. innocua* was quantified at T0 and T24, before and after a water rinse, by plating 1 mL of sample on OCLA.

The viability of *L. innocua* was assessed to account for potential environmental stress and the limitations of conventional culture methods in detecting sublethally injured cells. Viable bacteria were quantified using viability quantitative real-time PCR (v-qPCR), combined with ethidium monoazide (EMA) and propidium monoazide derivative (PMAxx), which selectively inhibit DNA amplification from non-viable cells (50). After staining, the samples were exposed to photolysis for 15 min using a PMA Lite LED system (Led Active Blue, GenIUL). Bacterial cells were then concentrated by centrifugation (9,000 × *g*, 4°C, 10 min), and the resulting pellet was stored at −20°C for DNA extraction. Genomic DNA was extracted using the DNeasy PowerSoil Pro kit (QIAGEN, Hilden, Germany) and quantified spectrophotometrically (NanoDrop ND-1000) using 260/280 nm and 260/230 nm absorbance ratios. Quantitative PCR targeting the 23S rDNA gene of the genus *Listeria* was performed on a QuantStudio 5 Real-Time PCR (Applied Biosystems, Waltham, MA, USA), following validated protocols (38). The standard curve and detection limit for *L. innocua* were established as previously described (50).

### Data analysis

The processing and analysis of the data were executed using R Statistical Software (version 4.4.0) (51), implemented within the integrated development environment RStudio (version 2024.12.1.563) (52). A Welch’s *t*-test was employed to assess the impact of box washing on the load of aerobic plate counts. The graphical representation of the data was made using *ggplot2* (version 3.5.1) (53) and *ggstatsplot* (version 0.13.0.9000) packages of R (54).

## Data Availability

Primary data and the R scripts used to generate the figures are available at http://hdl.handle.net/10261/404308. Raw sequence data for the isolates have been deposited in the European Nucleotide Archive (ENA) under project accession PRJEB101826.

## SUPPLEMENTAL MATERIAL

Supplemental tables aem.01396-25-s0001.docx Tables S1 and S2.

ASM does not own the copyrights to Supplemental Material that may be linked to, or accessed through, an article. The authors have granted ASM a non-exclusive, world-wide license to publish the Supplemental Material files. Please contact the corresponding author directly for reuse.
