# Supplementary material for: Environmental monitoring and equipment sanitation to mitigate Listeria risks in a soil-based controlled environment agriculture system
Source: Appl Environ Microbiol. 2025 Nov 20;91(12):e01396-25. doi: 10.1128/aem.01396-25 (PMC12724139; doi:10.1128/aem.01396-25)
Supplement: Supplemental tables — Tables S1 and S2. [file aem.01396-25-s0001.docx]

**Supplementary information**

**Table S1.** Characterization of *Listeria* isolates based on the presence of the *hly*, *iap*, and *sigB* genes and MALDI-TOF MS analyses. The table includes data from environmental monitoring sampling (EM), specifying the sampling site and isolate ID.

| **EM** | **Sampling site** | **Isolate ID** | ***hly*** | ***iap*** | ***sigB*** | **MALDI-TOF** |
| --- | --- | --- | --- | --- | --- | --- |
| 1 | Harvesting crates | 1 |  | positive | positive | *L. innocua* |
|  |  | 2 |  | positive | positive | *L. innocua* |
|  |  | 3 |  | positive | positive | *L. innocua* |
|  | Harvesting crates after vacuum | 1 |  | positive | positive | *L. innocua* |
|  |  | 2 |  | positive | positive | *L. innocua* |
|  |  | 3 |  | positive | positive | *L. innocua* |
|  | Columns | 1 |  | positive | positive | *L. innocua* |
|  |  | 2 |  | positive | positive | *L. innocua* |
|  |  | 3 |  | positive | positive | *L. innocua* |
|  |  | 4 |  | positive | positive | *L. innocua* |
|  |  | 5 |  | positive | positive | *L. innocua* |
|  |  | 6 |  | positive | positive | *L. innocua* |
|  | Boot covers | 1 | negative | positive | negative | *L. aquatica* |
| 2 | Harvesting crates | 1 | negative | positive | negative | *L. fleichmannii* |
|  | Harvesting crates after vacuum | 1 |  | positive | negative | NOIP |
|  |  | 2 |  | positive | negative | *L. grayi* |
|  |  | 3 |  | positive | negative | *L. aquatica* |
|  | Leaf vacuum machine | 1 |  | positive | negative | *Enterococcus faecalis* |
|  |  | 2 |  | positive | negative | *Enterococcus faecalis* |
|  |  | 3 |  | positive | negative | *Enterococcus faecalis* |
| 3 | Boot covers | 1 | positive | negative | positive | *Enterococcus faecalis* |
|  |  | 2 | positive | negative | positive | *Enterococcus faecalis* |
|  |  | 3 | positive | positive | positive | *L. monocytogenes* |

NOIP: No Organism Identification Possible

**Table S2**. Sampling sites and sequencing quality parameters for *Listeria* isolates, including total sequenced bases, read count, GC content (%), Q20 and Q30 quality metrics, percentage of mapped reads, and detected variants, compared to the reference genome of *Listeria monocytogenes* (GCF_000196035.1; 2,944,528 bp). These data correspond to the sequences after trimming the adapter sequences and the bases with a leading/tailing quality lower than three, as well as removing reads below 36 bases long.

| **Sampling sites** | **EM** | **Total bases (bp)** | **Total reads** | **GC (%)** | **AT (%)** | **Q20 (%)** | **Q30 (%)** | **Mapped Reads (%)** | **Variants** |
| --- | --- | --- | --- | --- | --- | --- | --- | --- | --- |
| Harvesting crates | 1 | 3,004,916,397 | 20,451,442 | 38.6 | 61.4 | 98.8 | 95.3 | 79.85 | 155,581 |
| Harvesting crates after vacuum | 1 | 1,775,389,981 | 12,274,798 | 38.6 | 61.4 | 98.5 | 94.2 | 77.15 | 153,405 |
| Column | 1 | 2,727,746,915 | 18,794,632 | 38.7 | 61.3 | 98.5 | 94.2 | 77.30 | 153,035 |
| Boot covers | 1 | 2,869,348,710 | 19,838,970 | 37.0 | 63.0 | 98.5 | 94.3 | 3.07 | 2,675 |
| Harvesting crates | 2 | 2,911,829,622 | 19,847,948 | 39.5 | 60.5 | 99.0 | 95.9 | 11.70 | 17,592 |
| Harvesting crates after vacuum | 2 | 2,677,643,558 | 18,540,102 | 41.4 | 58.6 | 98.5 | 94.0 | 10.26 | 12,576 |
| Harvesting crates after vacuum | 2 | 2,181,508,922 | 15,034,500 | 41.1 | 58.9 | 98.6 | 94.6 | 10.38 | 12,469 |
| Boot covers | 3 | 2,905,465,478 | 20,063,094 | 39.1 | 60.9 | 98.5 | 94.0 | 94.59 | 27,322 |

Sampling sites: Locations where *Listeria* isolates were collected.

Total Bases (base pairs, bp): Total number of bases sequenced.

Total Reads: Total number of sequencing reads. For Illumina paired-end sequencing, this value represents the sum of Read 1 and Read 2.

GC Content (%): Percentage of guanine-cytosine (GC) content in the sequenced data.

AT Content (%): Percentage of adenine-thymine (AT) content in the sequenced data.

Q20 (%): Percentage of bases with a Phred quality score greater than 20, indicating a base call accuracy of at least 99%.

Q30 (%): Percentage of bases with a Phred quality score greater than 30, indicating a base call accuracy of at least 99.9%.

Mapped Reads (%): Percentage of sequencing reads successfully aligned to the reference genome.

Variants: Number of genetic variations, including insertions, deletions, and single nucleotide polymorphisms (SNPs), identified in comparison to the reference genome.
